# A rapid on-line method for mass spectrometric confirmation of a cysteine-conjugated antibody-drug-conjugate structure using multidimensional chromatography

**DOI:** 10.1080/19420862.2015.1083665

**Published:** 2015-08-25

**Authors:** Robert E Birdsall, Henry Shion, Frank W Kotch, April Xu, Thomas J Porter, Weibin Chen

**Affiliations:** 1 Biopharmaceutical Business Operations, Waters Corporation; Milford, MA USA; 2 Pfizer Bioprocess Research & Development, Pfizer Inc; Pearl River, NY USA; 3 Pfizer Analytical Research & Development, Pfizer Inc; Pearl River, NY USA; 4 Pfizer Analytical Research & Development, Pfizer Inc; Andover, MA USA

**Keywords:** 2DLC, ADC, antibody-drug-conjugate, cysteine-drug-conjugate, drug-antibody-ratio, LC/LC/MS, multidimensional chromatography, positional isomers

## Abstract

Cysteine-conjugated antibody-drug conjugates (ADCs) are manufactured using controlled partial reduction and conjugation chemistry with drug payloads that typically occur in intervals of 0, 2, 4, 6, and 8. Control of heterogeneity is of particular importance to the quality of ADC product because drug loading and distribution can affect the safety and efficacy of the ADC. Liquid chromatography ultra-violet (LC-UV)-based methods can be used to acquire the drug distribution profiles of cysteine-conjugated ADCs when analyzed using hydrophobic interaction chromatography (HIC). However, alternative analysis techniques are often required for structural identification when conjugated drugs do not possess discrete ultra-violet absorbance properties for precise assessment of the drug-to-antibody ratio (DAR). In this study, multidimensional chromatography was used as an efficient method for combining non-compatible techniques, such as HIC, with analysis by mass spectrometry (LC/LC/QTOF-MS) for rapid on-line structural elucidation of species observed in HIC distribution profiles of cysteine-conjugated ADCs. The methodology was tested using an IgG1 mAb modified by cysteine conjugation with a non-toxic drug mimic. Structural elucidation of peaks observed in the HIC analysis (1^st^ dimension) were successfully identified based on their unique sub-unit masses via mass spectrometry techniques once dissociation occurred under denaturing reversed phase conditions (2^nd^ dimension). Upon identification, the DAR values were determined to be 2.83, 4.44, and 5.97 for 3 drug load levels (low-, medium-, and high-loaded ADC batches), respectively, based on relative abundance from the LC-UV data. This work demonstrates that multidimensional chromatography coupled with MS, provides an efficient approach for on-line biotherapeutic characterization to ensure ADC product quality.

## Abbreviations

ADC: antibody-drug conjugate
1D: 1^st^ dimension
2D: 2^nd^ dimension
BSM: binary solvent manager
CQA: critical quality attribute
DAR: drug-antibody-ratio
DTT: dithiothreitol
HIC: hydrophobic interaction chromatography
IgG: immunoglobulin G
LC-UV: liquid chromatography ultra-violet
mAb: monoclonal antibody
MP: mobile phase
MS: mass spectrometry
QSM: quaternary solvent manager
QTOF: quadrupole time-of-flight
RP: reversed phase
SDS-CE: sodium dodecyl sulfate-capillary electrophoresis
TCEP: (tris(2-carboxyethyl)phosphine)
TUV: tunable ultra-violet
UV: ultra-violet.

## Introduction

Antibody-drug conjugates (ADCs) represent a growing class of biotherapeutics currently being investigated for the treatment of cancer.^1-5^ The efficacy of ADCs, in part, is attributed to the underlying architecture of the conjugate, wherein a monoclonal antibody (mAb) is combined with a cytotoxin. The selectivity of the mAb toward over-expressed cell surface antigens associated with cancerous tumors facilitates the targeted delivery of a covalently linked cytotoxic agent, or “drug,” adjacent to the tumor surface. This therapeutic approach offers the selectivity of an antibody^6^ for targeted treatment of tumor cells while minimizing systemic toxicity effects from the highly potent drug.^2,7,8^ Successful launches of brentuximab vedotin (Adcetris®)^9^ and ado-trastuzumab emtansine (Kadcyla®)^3^ for the treatment of Hodgkin's lymphoma and breast cancer, respectively, illustrate the potential impact of these emerging biotherapeutic agents in cancer treatment. Engineering ADCs with preserved antibody binding activity and reproducible physicochemical properties that can be used as metrics during development and process control is highly desirable.^10,11^

**Figure 1.**
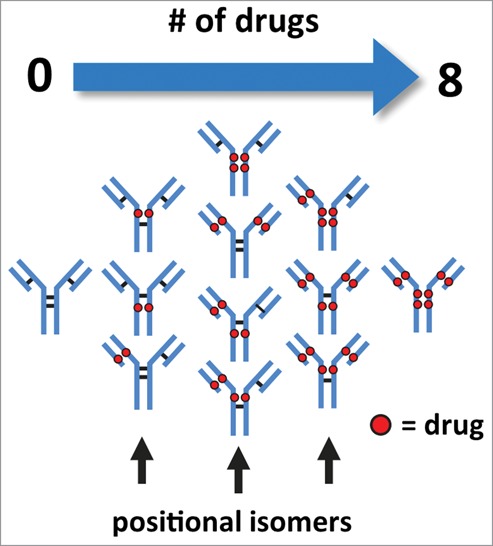
Illustration of cysteine-conjugated ADCs with various drug load distributions. Reduction of inter-chain disulfide bonds allows the conjugation of drugs through a maleimide-containing linker via the newly generated sulfhydryl groups. Conjugation of drugs via reduced inter-chain disulfide bonds generate ADCs with expected drug loads occurring in intervals of 2, 4, 6, and 8 with associated possible positional isomers.

Cysteine-conjugated ADCs are a sub-class of biotherapeutics that are manufactured with well-known conjugation chemistry. This type of ADC is typically less heterogeneous with respect to the quantity and distribution of drugs conjugated to the mAb^12^ in comparison to ADCs created via lysine-based conjugation methods.^13^ Control of heterogeneity is of particular importance as drug load and drug distribution can affect the efficacy, toxicology, and half-life/clearance properties of the ADC.^10,14,15^ In theory, the conjugation process produces a mixture of isoforms with the number of drugs conjugated in intervals of 0, 2, 4, 6, and 8 from the controlled reduction of the inter-chain disulfide bonds, each of which generates 2 sulfhydryl groups (**Fig. 1**).^12^ As a result, the reduced complexity of cysteine-conjugated ADCs allows for the use of liquid chromatography (LC)-based analytical methods such as hydrophobic interaction chromatography (HIC) to assess the heterogeneity of conjugated antibody.^15,6^

**Figure 2.**
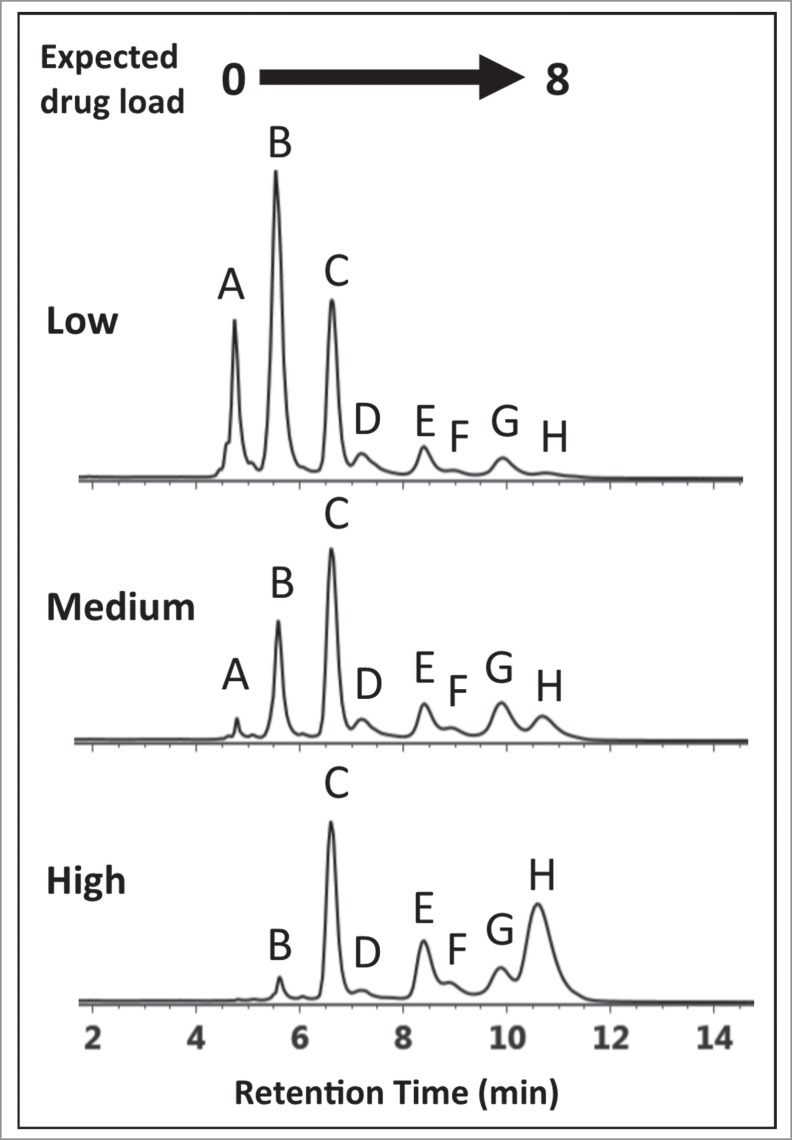
HIC chromatograms showing distribution profiles of cysteine-conjugated ADCs. Three batches of ADCs were synthesized, each with a different level of drug load (Low-, Medium-, and High-loaded) and analyzed using a 10 min gradient with a HIC column, 4.6 × 100 mm, 2.5 µm (see experimental). The distribution profiles exhibited multiple peaks that differed from expected profiles preventing unambiguous correlation of DAR 2, 4, 6, and 8 species with the peaks (B)–(H).

The conjugation process, when not entirely optimized, can present diagnostically different HIC distribution profiles (**Fig. 2**) that deviate from profiles containing the expected 5 peaks representing DAR values of 0, 2, 4, 6, and 8.^11,12,17^ In the absence of a reference standard or for conjugated drugs that do not possess discrete ultra-violet (UV) absorbance properties, shoulder peaks distributed throughout the chromatogram cannot be unambiguously assigned based on LC-UV methods alone.^12,15,16^ These peaks, which may represent ADC isoforms,^12,18^ incomplete drug conjugation,^17^ or post-translational modifications on the mAb,^19,20^ can affect the efficacy and safety^10,15^ of the ADC. Additional analysis is required for structural identification if precise assessment of drug loading and distribution are to be determined. For identification purposes, current LC-UV-based methods require the inclusion of an additional purification step followed by analysis using orthogonal techniques such as reversed phase (RP) LC or denaturing capillary electrophoresis (SDS-CE).^12,14,15,17^

In comparison, non-denaturing mass spectrometry (MS)-based methods have been adopted successfully in the intact analysis of ADCs, including the elucidation of sub-unit conjugated forms and site distribution of conjugated drugs.^21-25^ Recently, Valliere-Douglass et al. described a non-denaturing MS-based method for the determination of relative drug distribution in ADCs based on ion abundance and mass from the deconvoluted spectrum of cysteine-conjugated ADCs.^23^ A high degree of correlation in relative abundance for DAR species was observed when compared to traditional LC(HIC)-UV-based methods, despite the question raised on the possible ionization efficiency discrepancy of higher drug-conjugated species. Using a similar approach, Chen et al. improved ionization efficiency by enzymatically removing conjugated drug species while also observing a high degree of correlation with off-line LC(HIC)-UV methods.^22^ Innovative techniques that incorporate ion mobility (IM)-MS have also demonstrated their utility in the characterization of IgGs and ADCs.^25-29^ Debane and colleagues demonstrated the utility of native MS methods as well as IM-MS in the assessment of drug distribution in cysteine-conjugated ADCs.^25^ Similar to Valliere-Douglass et al. and Chen et al., their work found a high degree of correlation between semi-quantitative MS-based approaches and traditional HIC-UV-based methods. Interestingly, Debane et al. found that an offline HIC/native MS approach was able to detect the presence of unexpected odd-numbered conjugated species. The utility of MS-based techniques in the characterization of ADCs is firmly established in the literature. However, similar to LC-UV-based methods, additional steps involving enzymatic treatment are necessary for the elucidation of positional isoforms of cysteine-conjugated ADCs.^24,30^ Because this modality has the potential to redefine cancer treatment, rapid methods to identify ADC isoforms during biotherapeutic characterization are highly desirable.

Recent work has demonstrated multidimensional chromatography approaches to be effective in the characterization of impurities,^31^ glycopeptides,^32^ production excipients,^33^ and formulation degradants^34^ associated with therapeutic drugs. The straightforward experimental design associated with this methodology provides an elegant solution for the structural identification of peaks observed in LC-UV-based separations of ADCs, bypassing the need for sample preparation procedures such as enzymatic digests and manual fraction collection. As shown in **Table 1**, the coupling of 2 orthogonal separation dimensions increases the separation capacity in cysteine-conjugated ADC analyses and facilitates a method for structural elucidation of peaks observed in ADC HIC distribution profiles. In the 1^st^ dimension (y-axis), ADCs are separated by their hydrophobicity using established HIC techniques. Positional isoforms, which may be represented as additional peaks in the 1^st^ dimension separations (peaks (C)-(H), **Fig. 2**), can be identified by unique sub-unit masses (x-axis) via MS techniques once dissociation occurs under denaturing conditions. In this fashion, multidimensional chromatography renders an efficient method for combining otherwise non-compatible techniques, such as HIC and MS analysis (LC/LC/QTOF-MS) for rapid structural elucidation of species observed in HIC distribution profiles of cysteine-conjugated ADCs.^35,36^

**Table 1.** Multidimensional analysis. HIC separation in the 1^st^ dimension (y-axis) separates cysteine-conjugated ADCs based on their hydrophobicity associated with increasing DAR species. When subjected to an orthogonal 2^nd^ dimension (x-axis) separation such as RPLC-MS, cysteine-conjugated ADCs dissociate into their respective sub-units due to denaturation by the mobile phases and temperature employed. The discrete masses generated from the unique sub-units for each conjugated species facilitate structural identification of positional isoforms associated with cysteine-conjugated ADCs
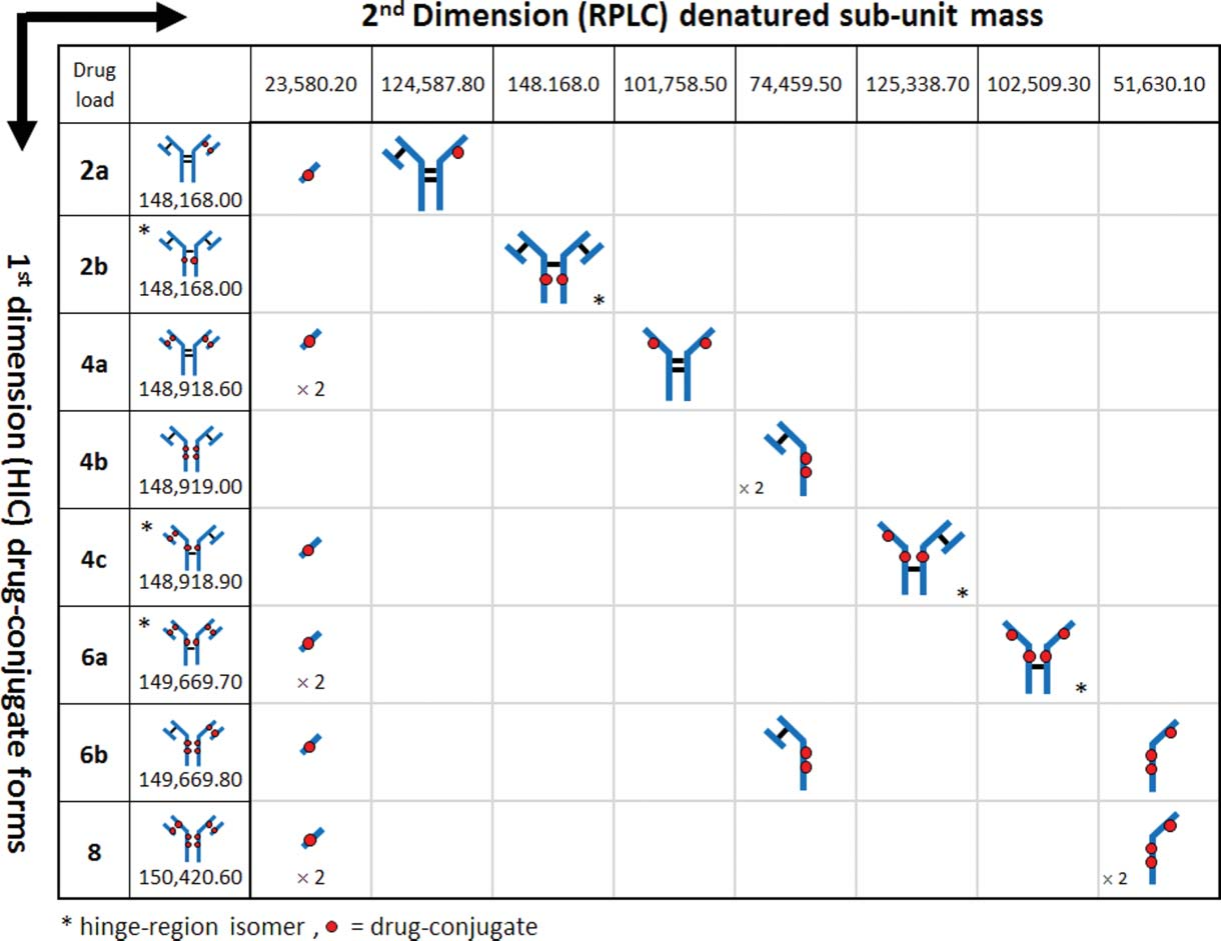

**Table 2.** Drug-to-antibody ratio determination. Individual DAR contributions for each drug loaded species (e.g., DAR 0, 2, 4, 6, 8) was calculated based on the sum relative peak area for the distribution species denoted by the dashed lines in **Figure 6** The average DARs were determined to be 2.83, 4.44, and 5.97 for the low-, medium-, and high-loaded ADC batches, respectively

	Drug load																
N=3	0			2			4			6			8				
Batch	Area (%)	RSD (%)	DAR contribution	Area (%)	RSD (%)	DAR contribution	Area (%)	RSD (%)	DAR contribution	Area (%)	RSD (%)	DAR contribution	Area (%)	RSD (%)	DAR contribution	Total DAR	RSD (%)
Low	16.73	0.28	0	40.71	0.23	0.81	28.56	0.24	1.14	12.54	0.50	0.75	1.50	10.11	0.12	2.83	0.38
Med	2.80	0.34	0	18.85	0.46	0.38	41.69	0.22	1.67	26.87	0.13	1.61	9.79	1.69	0.78	4.44	0.20
High	0.37	1.45	0	3.42	0.14	0.07	30.67	0.18	1.23	28.60	0.32	1.72	36.90	0.37	2.95	5.97	0.06

Experimental results from an analysis of 3 batches of an ADC at 3 different DARs by the 2D-LC/QTOF method are presented here. The method preserves intact ADC characterization information while facilitating the online fractionation and identification of peaks under investigation from cysteine-conjugated ADC HIC distribution profiles. The unambiguous identification of peaks associated with distribution profiles in HIC separations of cysteine-conjugated ADCs increase the confidence for accurate determination of DAR and ADC profiles. The results demonstrate that the 2D LC/MS method is well suited for rapid analysis of ADC isoforms for the development and characterization of ADCs.

## Results

An IgG1 mAb was modified by cysteine conjugation to a non-toxic drug that mimics the hydrophobicity of a cytotoxic drug to give ADC batches at 3 DARs and analyzed by HIC chromatography to assess drug distribution profiles. As seen in **Figure 2**, multiple peaks were observed in all 3 ADC batches presumably representing individual DARs of 0, 2, 4, 6, and 8. Based on the underlying separation mechanism of HIC, as well as knowledge of the possible isoforms that could be formed by cysteine conjugation using a partial reduction/conjugation process, it was assumed that the elution order was associated with increasing DAR species. The identity of peak (A) was readily confirmed experimentally using the retention time alignment of the unconjugated mAb in a separate HIC separation (supplemental material). The trending reduction in intensity of peaks (A–C) combined with the increasing intensity of peaks (E) and (H) across the low- to high-loaded ADC batches suggests that these peaks represent individual ADC DAR species of 0, 2, 4, 6, and 8, respectively.^15,16^ However, assignment of peaks (D)-(H) based on distribution profiles and intensity trends from LC-UV data is challenging considering that conjugation conditions, mAb identity, and the presence of isoforms can affect ADC distribution profiles. To determine if unambiguous peak identification and improved confidence in determining critical quality attributes^11^ (CQAs) such as individual and total DAR values could be achieved, an MS-based approach was evaluated.

Mass confirmation of peaks observed in ADC HIC distribution profiles would provide a method for determining the individual DAR values for ambiguous peaks, e.g., (F)-(G) in **Figure 2**. However, the high concentration and low volatility of the salts used in HIC separations are not compatible with direct MS analysis.^35,36^ Coupling LC (HIC)-UV to RPLC-MS with selective sampling of LC (HIC)-UV profiles would render an efficient online method for the assessment of CQAs associated with unassigned peaks. In contrast to non-denaturing MS methods, the denaturing conditions used in traditional RPLC, when coupled to HIC, provides a means to remove non-volatile buffer components while simultaneously dissociating ADCs into their respective non-covalently-linked sub-units for identification of isoforms by mass. Such information is currently not directly reflected with non-denaturing MS methods. To this end, an on-line multidimensional chromatography approach was used for elucidating the peak identities.

**Figure 3.**
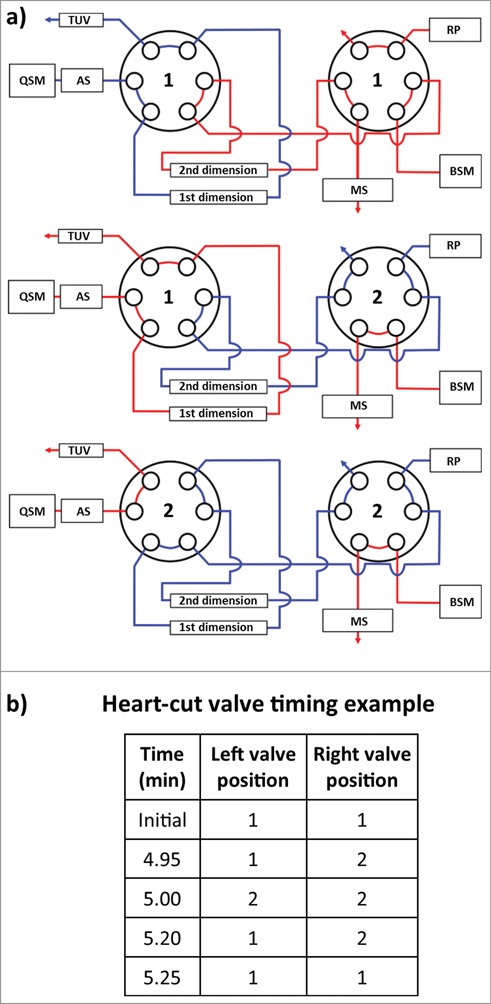
Instrument configuration schematic. (**A**) A column manager housing two 6-port 2-position valves was configured as illustrated by the schematic to perform heart-cuts of the 1^st^ dimension separation. Valve position is denoted numerically as position 1 and 2. Abbreviations are defined as QSM: quaternary solvent manager, AS: auto sampler, TUV: tunable ultraviolet detector, BSM: binary solvent manager, RP: regenerative pump, MS: mass spectrometer. (**B**) Heart-cuts were performed in 0.2 minute intervals at or near the apex of the peak under investigation. An example of the heart-cut being performed on peak (**C**) from **Figure 5c** is illustrated in the example. The heart-cut was bracketed with a 3 second interval to purge residual ammonium sulfate in the fluidic path post 1^st^ dimension column.

Coupling of orthogonal analytical techniques (HIC with RPLC/QTOF-MS) was achieved through the use of two 6-port, 2-position valves housed in a column manager (**Fig. 3**). Fractions (or “heart-cuts”) from peaks of interest observed in the first dimension HIC separation were selected by coordinated timed switches between the 2 valves so that protein species contained in the HIC fraction could be transferred from the 1^st^ dimension column onto the 2^nd^ dimension column. Subsequent separation on the 2^nd^ dimension column (and MS detection) allows the protein species to be identified.

**Figure 4.**
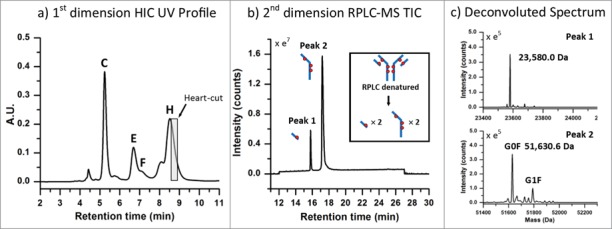
ADC analysis using an LC(HIC)/LC(RP)/QTOF-MS approach. (**A**) A 0.20 min heart-cut (100 µL) was initiated post-apex on the latest eluting peak from the HIC separation (peak (H)) of the high cysteine-conjugated ADC batch. (**B**) The transferred fraction was desalted and separated using a 15 min gradient by RPLC with expected dissociated sub-units shown in the inset. (**C**) MS spectra were deconvoluted and determined to be indicative of the light-chain (23,580.0 Da) and heavy-chain (51,630.6 Da) containing 1 and 3 drugs, respectively.

To test the validity of this approach, a separation of the latest-eluting species observed in the HIC distribution profile, which represents the highest DAR species, was targeted for experimentation. To maximize the amount of protein transferred from higher-loaded species (peaks (C)-(H)) to the 2^nd^ dimension column in a relatively small volume, the high loaded ADC batch (**Fig. 2**) was selected for this and subsequent analyses. To ensure column pressure tolerances were not exceeded when both columns were engaged in the fluidic path, a relatively short HIC column (4.6 × 35 mm, 2.5 µm) was employed. Peak profiles were assessed and deemed sufficiently comparable to the profiles observed with the 100 mm version (**Fig. 2**) without the need to scale the method for the shorter column. As shown in **Figure 4a**, a 0.20 min heart-cut was programmed to transfer a 0.100 mL fraction of the late-eluting species (peak (H)) from the HIC separation (visualized by the gray box) to the 2^nd^ dimension RP (C_4_) column. The heart-cut was performed post-peak apex to ensure that relatively more homogeneous species were selected and transferred to the 2^nd^ dimension analysis. Desalting of the sample on the RP column, which facilitated coupling of a method that is normally not compatible with MS instrumentation, was performed using a high percentage of aqueous component of the 2^nd^ dimension RPLC mobile phases (100% water containing 0.1% formic acid) with the MS-valve position set to waste. As shown in **Figure 4b**, the subsequent RPLC separation of the fraction of interest resulted in 2 peaks, which represent the dissociated sub-units of the fractionated species. Deconvolution of the raw mass spectrum for each peak is shown in **Figure 4c**. From the deconvoluted spectrum, a mass for the early eluting peak was found to match the predicted mass for a light-chain conjugated to one drug (23,580.0 Da, mass error −8.48 ppm) and masses for the late eluting peak were found to be consistent with the heavy-chain (G0F/G1F glycoforms) with 3 drugs (51,630.6 Da, mass error +9.97 ppm). As shown in **Table 1**, these 2 masses can only be observed for the DAR 8 ADC species, since the 2 dissociated sub-unit peaks in the 2^nd^ dimension separation represent a corresponding sub-unit pair (**Fig. 4b** inset). With mass confirmation of peak (H) as the DAR 8 ADC species, and no higher-loaded species observed across the low-, medium-, and high-loaded ADC samples, the remaining peaks must have DAR values < 8. A complete list of MaxEnt1 parameters and mass shifts can be found in the supplemental material.

**Figure 5.**
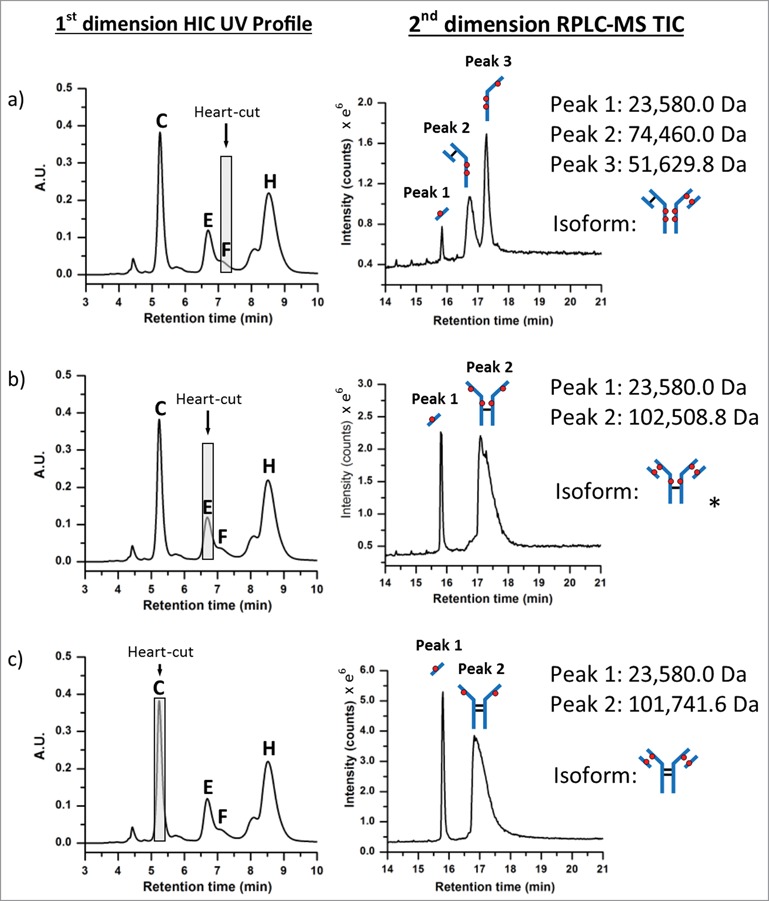
Positional isoform identification using HIC/RP/QTOF-MS. (**A**) A 0.20 min heart-cut fraction of peak (**F**) dissociated into 3 unique sub-unit masses under RPLC conditions which corresponded to an ADC with 6 drugs (DAR 6) as illustrated by the isoform structure provided. (**B**) A 0.20 min heart-cut fraction of peak (**E**) was also determined to be an isomer of DAR 6 with the corresponding structure shown in the illustration. (**C**) A 0.20 min heart-cut of peak (**C**) was confirmed to be an ADC bearing 4 drugs with both light-chain and their corresponding heavy-chain sulfhydryl sites occupied with drugs as shown in the illustration.

Using the same experimental approach, peaks ((C), (E), and (F)) were investigated (**Fig. 5**) using 0.20 min heart-cuts centered at the apex of the target peak. Fractionation of peak (F), as shown in **Figure 5a**, resulted in 3 peaks in the 2^nd^ dimension separation. The deconvoluted masses associated with each peak could only arise from the positional isoform represented by the 6-loaded ADC species (i.e., 6b in **Table 1**). Peak (E), as shown in **Figure 5b**, was determined to be the DAR 6 isomer 6a (see **Table 1**). Meanwhile, analysis of peak (C) confirmed the identity to be the DAR 4 isomer 4a (**Fig. 5c**, **4a**, **Table 1**). A mass difference of ∼ 17 Da was observed for 2^nd^ dimension peak 2 from HIC peak (C). This mass loss is likely the result of dehydration of the fragment (i.e., loss of one water molecule), which can occur in the mass spectrometer, HIC, or RP-HPLC environments, or originate in the native conjugate; localization of which requires further studies. However, isoform assignment was not impeded due to the contrast in mass shifts associated with the sub-unit forms shown in **Table 1**, affirming the robustness of such an approach for peak assignment of cysteine-conjugated ADCs.

**Figure 6.**
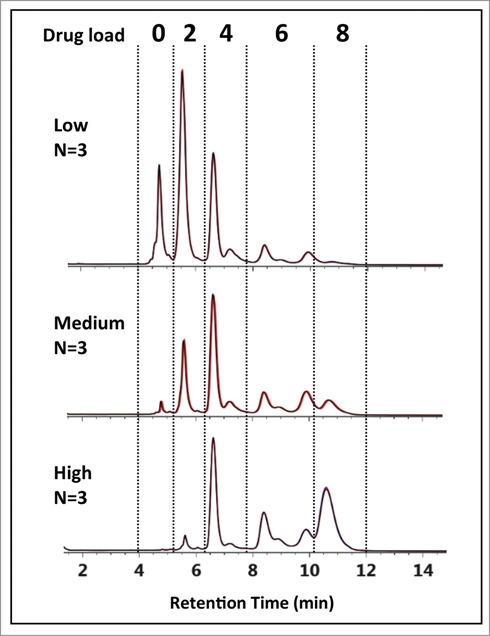
HIC-UV assessment of drug load distribution. Using peak identification determined from the multidimensional chromatography-MS method, drug load distribution was assessed in triplicate for the 3 batches of cysteine-conjugated ADCs. Overlays of the chromatograms (black, red, blue) are shown to demonstrate the reproducibility and robustness of the methodology.

Interestingly, peak (C) appears to be the major DAR 4 species across all 3 ADC batches based on peak intensities in **Figure 2**, suggesting a kinetically more favorable reaction product compared to the other DAR 4 ADC isomers.^12,37^ In addition, for the DAR 6 ADC species, the intensities of peak (F) and peak (G) are lower than that from peak (E) across all 3 samples. Closer inspection of relative peak area indicated peak (F) at 14.1% and peak (G) at 42.2% maintained similar peak area with respect to peak (E) at 43.7% across the 3 samples. The results suggest that the accessibility of the hinge-region cysteine residues plays a critical role in reaction kinetics of cysteine drug-conjugation chemistry, and conjugation of thiol groups from the cysteine residues that form the light chain–heavy chain disulfide bond is a preferred pathway for this IgG1 mAb under the reduction/conjugation conditions employed. The existence of 2 inter-chain disulfide bonds in the hinge region of all IgG1 molecules suggests that peak (E) may represent either one of the isomers or both isomers. The confirmation of which individual isomer exist (or both) would require additional experiments involving purification and enrichment of the peak, followed by enzymatic treatment for analysis,^30^ which is beyond the scope of the current work. Nonetheless, unambiguous identification of the major HIC peaks of cysteine-conjugated ADCs was achieved using the multidimensional chromatography approach. With the additional peaks being identified as ADC isoforms, drug distribution profiles could be readily assigned across all 3 cysteine-conjugated ADC batches as shown in **Figure 6**. With this knowledge, DAR values based on relative abundance from the LC-UV data were determined to be 2.83, 4.44, and 5.97 for the low-, medium-, and high-loaded ADC batches, respectively (**Table 2**).

## Discussion

Targeted immunoconjugate-based therapies such as ADCs offer the potential to redefine our understanding and approach to the treatment of cancer. Cysteine-conjugated ADCs using conventional reduction/alkylation.^12^ represent a sub-class of ADCs that offer reduced complexity that are readily characterized using LC-UV-based techniques. Accurate assessment of CQAs such as ADC drug distribution and associated DAR values, which can affect efficacy and safety, is critical for the research and development of ADCs. Development of immunoconjugate therapies with diagnostically different chromatographic profiles that deviate from expected profiles requires the use of orthogonal techniques that augment existing characterization methods, which allows accurate assessment of CQAs. The utility of MS-based techniques to gain additional insight in the characterization of biotherapeutics has been well established in literature.^21-29^ However, techniques that are incompatible with MS analysis are often encountered in the characterization of ADCs such as in the case of HIC-UV, which is frequently used to assess drug distribution and drug load attributes.^11^ Efficient methods that complement existing characterization techniques and can be readily adapted into the biopharmaceutical production environment are highly desirable.

This work has demonstrated that multidimensional chromatography renders a viable interface for the hyphenation of normally non-compatible methods with MS instrumentation. The 2D methodology presented here is particularly useful in providing rapid characterization of positional isoforms associated with conventional cysteine ADCs based on their unique sub-unit structures as well as DAR determinations. It is also applicable in general to characterization of HIC peaks for ADCs with other conjugation chemistries, including site-specific cysteine conjugation,^10,38,39^ although information obtained may be tailored to the specific chemistry used for conjugation.

## Material and Methods

Chemicals and reagents were purchased from Sigma Aldrich unless otherwise stated. MS-grade solvents were used for mobile phase preparation. Aqueous buffers were prepared with water purified using a lab water filtration system (Millipore).

### ADC Stock Solutions

ADCs were prepared according to conventional reduction protocols.^12^ To show the proof of concept and for ease of handling, a noncytotoxic drug-mimic was selected as the drug for this study. Tris(2carboxyethyl)phosphine hydrochloride (TCEP) was added to a solution of the IgG1 mAb in phosphate-buffered saline (pH 7.2) to reduce the inter-chain disulfide bonds. The maleimide-containing drug-mimic (C_23_H_24_N_2_O_3_, MW = 376.46 Da) was then added and allowed to react. TCEP and drug-mimic concentrations were controlled to produce 3 stock samples with increasing DAR (low-, medium-, and high-loaded samples). Following the conjugation reaction, the ADC mixtures were buffer-exchanged into 20 mM histidine buffer (pH 6.0) and adjusted to a concentration of ∼ 10.0 mg/mL. An unconjugated mAb stock was also prepared at ∼ 10.0 mg/mL. The stock solutions were stored at −80°C prior to use.

### Sample Preparation

HIC samples were prepared at a concentration of 2.0 mg/mL in 1M (NH_4_)_2_SO_4_ using a 62.5mM sodium phosphate buffer, pH 6.7. Analyses were performed on a 10.0 µL injection volume. A reversed phase chromatography control sample was prepared through partial reduction of the unconjugated mAb. Briefly, a 1.0 mg/mL solution of unconjugated mAb prepared in 25mM phosphate buffer, pH 7.8, was reduced in 1 mM dithiothreitol (DTT) at 37°C for 20 min. An equal amount of sample was mixed with 0.1% formic acid (v/v) in purified water to give a final concentration of 0.5 mg/mL.

### Chromatography

An ACQUITY H-Class Bio equipped with a commercially available 2D technology configuration (Waters Corp.) was used for the experiments. **Figure 3A** is a schematic of the instrument setup denoting column, pump, and plumbing configuration. Mobile phase (MP) reservoirs for the 1^st^ dimension quaternary solvent manager (QSM) were prepared as follows: MP A: 2.5 M (NH_4_)_2_SO_4_ in 125 mM sodium phosphate buffer, pH 6.7, MP B: 125 mM sodium phosphate buffer, pH 6.7, MP C: n-propanol, MP D: 18 MΩ purified water. A 4.6 × 35 mm, 2.5 µm n-butyl HIC column (Waters Corp.) was conditioned with the unconjugated mAb until the integrated area and retention time stabilized using a 10 min gradient from 1.25 M (NH_4_)_2_SO_4_, in 65 mM sodium phosphate buffer, pH 6.7, 5% n-propanol (50% MP A : 0% MP B : 5% MP C : 45% MP D) to 65 mM sodium phosphate buffer, pH 6.7, 5% n-propanol (0% MP A : 50% MP B : 5% MP C : 45% MP D). Mobile phase conditions were held constant for 5 min at the end of each gradient followed by 15 min of column reconditioning at initial conditions. Column temperature and flow rate were set at 25°C and 0.500 mL/min, respectively.

Mobile phase reservoirs for the 2nd dimension binary solvent manager (BSM) were prepared as follows: MP A: aqueous solution containing 0.1% formic acid v/v, MP B: acetonitrile containing 0.1% formic acid v/v. A 2.1.mm × 50.mm, 1.7 µm reversed-phase C_4_ column (BEH C_4_, Waters Corp.) was conditioned with the reduced unconjugated mAb in a 1D configuration until the integrated area and retention time stabilized. For RP separations, fractions of interest were transferred from the 1^st^ dimension HIC column to the 2^nd^ dimension C_4_ column using a regenerative pump with the left and right valves set in position 2 (**Fig. 3a**). Once transferred, a 12 min gradient from 100% A to 80% A was performed to stack and de-salt the fraction of interest using the binary solvent manager. After the 12-min desalting period, a 15 min separation gradient was performed from 80% to 50% MP A. At the end of the gradient, a 3 min column reconditioning step was performed at initial conditions. Column temperature and flow rate were set at 80°C and 0.500 mL/min, respectively.

Transfer of fractions of interest or “heart-cuts” from the 1^st^ dimension column to the 2^nd^ dimension column were programmed through the events tab using the column manager control interface. Valve position timing is illustrated in **Figure 3b** and were adjusted for each peak apex under investigation. A regenerative pump delivering an isocratic gradient of MP B from the 1^st^ dimension separation at 0.500 mL/min was used to perform the heart-cut and to reduce the amount of residual ammonium sulfate in the fluidic path post 1^st^ dimension column. A 0.05 min delay was programmed at the beginning and end of each heart-cut to purge the fluidic path of residual ammonium sulfate using 50% MP B : 5% MP C : 45% MP D from the 1^st^ dimension reservoirs. A tunable ultra-violet (TUV) detector (Waters Corp.) equipped with a 5-mm titanium flow cell was incorporated post the 1^st^ dimension separation to monitor the heart-cut procedure. Single wavelength detection was performed at an A_max_ of 220 nm with a sampling rate of 20 Hz.

### Ms settings

A quadrupole time-of-flight mass spectrometer (Xevo G2 QTof, Waters Corp.) was used for MS analysis post 2^nd^ dimension column (**Fig. 3a**). Continuum data were acquired in sensitivity mode with positive polarity. A mass range from 500 to 4,000 m/z was used for data collection, and the MS data was only collected from 12 to 27 min as defined in the chromatography section. The flow was directed to waste via the MS valve event manager when the 2D system was operated outside the acquisition time window. Additional instrument settings were set as follows: capillary voltage 3.00 kV, sample cone, 80.0 V, extraction cone, 4.0 V, source temperature 100°C, desolvation temperature 350.°C, and desolvation gas flow 600 L/Hr. Data from the MS analysis for the ADC subunits and the light chain and heavy chain of native mAbs were processed by MaxEnt 1 algorithm within MassLynx. Typically twenty iterations of MaxEnt 1 deconvolution were performed on the raw spectrum, and a mass accuracy error ≤10 ppm was achieved for the light-chain and non-deglycosylated heavy-chain. Individual parameter settings can be found in the supplemental material.
