# Supplementary material for: A rapid on-line method for mass spectrometric confirmation of a cysteine-conjugated antibody-drug-conjugate structure using multidimensional chromatography
Source: MAbs. 2015 Aug 25;7(6):1036–44. doi: 10.1080/19420862.2015.1083665 (PMC4966495; doi:10.1080/19420862.2015.1083665)
Supplement: Supplemental_material.pdf [file kmab-07-06-1083665-s001.pdf]

Supporting Information:

1st dimension (HIC)	2nd dimension (RPLC)	Structure	MaxEnt 1 Mass range	MaxEnt 1 resolution	MaxEnt 1 width	MaxEnt 1 Iterations	Theoretical mass (Da)	deconvoluted mass (Da)	Mass error (Da)
**Reduced un-conjugated mAb**	Peak 1	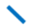	20,000:30,000	0.20	0.70	20	23,205.8	23,205.8	0.0
	Peak 2	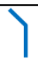	49,500:51,500	0.20	2.10	20	50,506.8	50,506.6	-0.2
**Peak (h)**	Peak 1	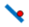	20,000:30,000	0.20	0.60	20	23,580.2	23,580.0	-0.2
	Peak 2	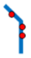	50,500:53,000	0.20	2.00	20	51,630.1	51,630.6	0.5
**Peak (f)**	Peak 1	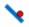	20,000:30,000	0.20	0.50	15	23,580.2	23,580.0	-0.2
	Peak 2	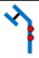	73,000:76,000	0.20	0.70	20	74,459.5	74,460.0	0.5
	Peak 3	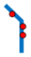	50,500:53,000	0.20	0.70	20	51,630.1	51,629.8	-0.3
**Peak (e)**	Peak 1	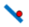	20,000:30,000	0.20	0.60	20	23,580.2	23,580.0	0.2
	Peak 2	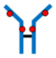	102,000:103,500	0.20	2.35	20	102,509.3	102,508.8	-0.5
**Peak (c)**	Peak 1	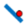	20,000:30,000	0.20	0.65	20	23,580.2	23,580.0	-0.2
	Peak 2	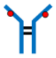	101,150:102,500	0.20	2.30	20	101,758.5	101,741.6	-16.9

Table S1. Deconvolution Parameters and Results. Instrument performance was found to be acceptable with the deconvolution of partially reduced unconjugated IgG1 mAb control resulting in two observed peaks in the RPLC analysis corresponding to the light chain and heavy chain of the mAb. The mass accuracy was determined to be within ±10.0 ppm. HIC drug distribution fractions (1st dimension) and associated sub-units (2nd dimension) along with their corresponding MaxEnt1 settings are listed for the peaks under analysis in this study.

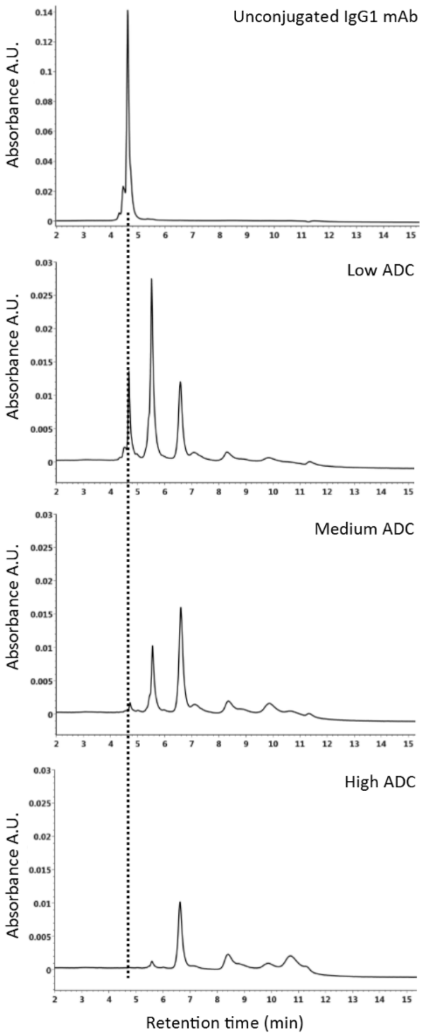

Figure S1. Time Alignment of Cysteine-conjugated ADC Batches. Cysteine-conjugated ADC batches with increasing level of drug loading were separated using a HIC column (4.6 × 100 mm, 2.5 μm) and compared to the unconjugated IgG1 mAb. Through time-alignment of the chromatograms, the peak associated with the unconjugated mAb was readily identified in the low- and medium-loaded ADC batches and at trace levels in the high-loaded ADC batch.
